# Cerebral Hemodynamics and Microvasculature Changes in Relation to White Matter Microstructure After Pediatric Mild Traumatic Brain Injury: An A-CAP Pilot Study

**DOI:** 10.1089/neur.2022.0050

**Published:** 2023-01-24

**Authors:** Andrew P. Lapointe, Ashley L. Ware, Chris C. Duszynski, Antonia Stang, Keith Owen Yeates, Jeff F. Dunn

**Affiliations:** ^1^ Department of Radiology, Cumming School of Medicine, Experimental Imaging Centre, University of Calgary, Calgary, Alberta, Canada.; ^2^ Alberta Children's Hospital Research Institute, Calgary, Alberta, Canada.; ^3^ Hotchkiss Brain Institute, University of Calgary, Calgary, Alberta, Canada.; ^4^ Department of Psychology, University of Calgary, Calgary, Alberta, Canada.; ^5^ Department of Psychology, Georgia State University, Atlanta, Georgia, USA.; ^6^ Department of Neurology, University of Utah, Salt Lake City, Utah, USA.; ^7^ Department of Pediatrics, University of Calgary, Calgary, Alberta, Canada.

**Keywords:** diffusion tensor imaging, fractional anisotropy, functional near-infrared spectroscopy, mean diffusivity, multi-modal imaging, pediatric mild traumatic brain injury

## Abstract

Advanced neuroimaging techniques show promise as a biomarker for mild traumatic brain injury (mTBI). However, little research has evaluated cerebral hemodynamics or its relation to white matter microstructure post-mTBI in children. This novel pilot study examined differences in cerebral hemodynamics, as measured using functional near-infrared spectroscopy (fNIRS), and its association with diffusion tensor imaging (DTI) metrics in children with mTBI or mild orthopedic injury (OI) to address these gaps. Children 8.00–16.99 years of age with mTBI (*n* = 9) or OI (*n* = 6) were recruited in a pediatric emergency department, where acute injury characteristics were assessed. Participants completed DTI twice, post-acutely (2–33 days) and chronically (3 or 6 months), and fNIRS ∼1 month post-injury. Automated deterministic tractography was used to compute DTI metrics. There was reduced absolute phase globally and coherence in the dorsolateral pre-frontal cortex (DLPFC) after mTBI compared to the OI group. Coherence in the DLPFC and absolute phase globally showed distinct associations with fractional anisotropy in interhemispheric white matter pathways. Two fNIRS metrics (coherence and absolute phase) differentiated mTBI from OI in children. Variability in cerebral hemodynamics related to white matter microstructure. The results provide initial evidence that fNIRS may have utility as a clinical biomarker of pediatric mTBI.

## Introduction

Pediatric mild (mTBI) traumatic brain injury (TBI) is a highly prevalent global public health concern.^1,2^ No robust biomarker is available for clinical detection or prognostication of mTBI,^3^ although advanced neuroimaging techniques show promise in addressing this important knowledge gap. Diffusion tensor imaging (DTI) can detect alterations in white matter microstructure in both early and late phases after pediatric mTBI.^3,4^

In contrast, little is known about functional near-infrared spectroscopy (fNIRS) for detecting differences in cerebral hemodynamics in pediatric mTBI. Yet, fNIRS, a relative measurement of oxy- and deoxyhemoglobin, has shown promise as a novel biomarker of brain injury.^5–7^ Lower coherence, a measure of the variability of time differences between two time series in a specific frequency band, have been noted after mTBI, with most changes demonstrated within the dorsolateral pre-frontal cortex (DLPFC).^8^

Growing evidence indicates that combining information from multiple imaging modalities can increase both the accuracy and reliability of classifying clinical populations.^9–13^ Knowledge gained through multi-modal neuroimaging studies may help to bridge gaps in current knowledge and increase the understanding of how mTBI affects brain tissue and functioning.^14^

This study aimed to examine cerebral hemodynamics and its relationship to white matter microstructure in children with mTBI or mild orthopedic injury (OI). To date, no previous study has utilized both fNIRS and DTI to study pediatric mTBI. We compared injury groups on fNIRS metrics roughly 1 month post-injury. We hypothesized that changes observed in fNIRS may relate to changes in white matter microstructure subcortically, as measured with DTI.

## Methods

This was a substudy of the Calgary cohort of the A-CAP (Advancing Concussion Assessment in Pediatrics) study, a large multi-site study that used a prospective, concurrent cohort design to study outcomes longitudinally in pediatric mTBI.^15^ Children were between 8.00 and 16.99 years of age. Within the Calgary cohort of A-CAP, 226 children completed DTI scans at two time points post-injury. This substudy included DTI data from 15 children who also completed fNIRS at ∼1 month post-injury, 9 with mTBI (age mean [*M*] = 13.41 years, standard deviation [*SD*] = 2.09; 4 females) and 6 with OI (age *M* = 12.18 years, *SD* = 3.09; 4 females).

For both groups, acute injury signs and symptoms were assessed within 48 h post-injury, at the time of enrollment in the emergency department at Alberta Children's Hospital. All eligible participants (i.e., without magnetic resonance imaging [MRI] contraindication; see details below) completed 3 Tesla MRI without sedation at a post-acute assessment (i.e., targeted for 10 days post-injury; range, 2–33) and were randomly assigned to complete a chronic MRI scan at 3 or 6 months post-injury. A subset of the children recruited at the Calgary site also completed fNIRS at ∼1 month post-injury.

This study was conducted with the approval of the conjoint health research ethics board at the University of Calgary, and all participants provided informed assent, when appropriate, and parents or guardians provided written informed consent. The A-CAP study protocol is published^15^; the specific methodology for the present study is described in detail below.

### Inclusion/exclusion criteria

Children with mTBI were included if they sustained a TBI resulting in at least one of the following criteria, consistent with the World Health Organization^16^ definition of mTBI: an observed loss of consciousness, a Glasgow Coma Scale score of 13 or 14, or at least one acute sign or symptom of concussion as noted by emergency department medical personnel on a standard case-report form.^16–18^ Children were excluded if they demonstrated delayed neurological deterioration (i.e., Glasgow Coma Scale <13), required neurosurgical intervention, or had loss of consciousness >30 min or post-traumatic amnesia >24 h.

Children with OI were included if they sustained an upper or lower extremity fracture, sprain, or strain because of blunt force/physical trauma associated with an Abbreviated Injury Scale score ≤4.^19^ Children were excluded from the OI group if they had any TBI or signs or symptoms of concussion at the time of recruitment, or any injury requiring surgical intervention or procedural sedation.

Exclusion criteria for both injury groups included any other severe injury; previous concussion within the past 3 months; hypoxia, hypotension, or shock during or after the injury; history of previous TBI or severe psychiatric disorder requiring hospitalization; pre-morbid neurological disorder or intellectual disability; injury resulting from non-accidental trauma; or any MRI contraindications. Additional inclusion/exclusion criteria are described in the published study protocol.^15^

### Functional near-infrared spectroscopy

Participants completed fNIRS (NIRScout; NIRx Medical Technologies, Los Angeles, CA) at rest and during a visuospatial 2-back working memory (six blocks) task at two near-infrared wavelengths of 760 and 850 nm, with a sampling frequency of 3.96 Hz using a custom array over the bilateral DLPFC and motor cortices. Assessments were completed ∼36 days post-injury (*M* = 35.73, *SD* = 5.02). At this time point, the Health and Behavior Inventory^20^ was completed by the child, and somatic (OI = 3.25 ± 3.77; mTBI = 3.71 ± 5.35) and cognitive (OI = 4.75 ± 8.22; mTBI = 7.86 ± 9.28) scores were computed (shown as mean and standard deviation).

### Diffusion tensor imaging

MRI acquisition and processing are described elsewhere.^19^ Briefly, after image pre-processing and quality assurance procedures,^19^ automated deterministic tractography was performed using the open-source Automated Fiber Quantification software package (v1.2)^21^ using the default parameters.^22^ Non-linear transformation was used to apply a waypoint region-of-interest template to individual images in native (diffusion-weighted) space to identify the forceps major (splenium) and forceps minor (genu) of the corpus callosum, along with five major white matter tracts in each hemisphere: corticospinal tract, inferior fronto-occipital fasciculus, superior longitudinal fasciculus, thalamic radiations, and uncinate fasciculus. Identified tracts were visually inspected for accuracy.

Because of an unequal representation of sex between groups in our subsample, we normalized DTI data obtained to the larger Calgary cohort of the A-CAP data set. This was completed by first computing the difference between the chronic and post-acute values in both DTI metrics. Subsequently, we computed a z-score for each DTI metric from our subset within the larger distribution (*n* = 226). This normalizes the data to a larger distribution and reduces potential bias in our small sample size.

### Statistical analyses

#### Part 1: Group differences in functional near-infrared spectroscopy metrics

Statistical analyses were completed in RStudio (v1.4.1106)^23^ with alpha levels of 0.05 for all tests. fNIRS measures were averaged over all connections before subsequent analyses. Linear models were performed to evaluate group differences for each fNIRS metric (absolute phase and coherence) during two conditions (rest and 2-back). A second analysis evaluated changes in coherence specifically in the DLPFC given previous evidence describing changes in this area.^8^

#### Part 2: Relationship between functional near-infrared spectroscopy and diffusion tensor imaging metrics

fNIRS metrics that differed significantly between groups were subsequently examined as separate dependent variables in general linear models with normalized mean diffusivity (MD) or fractional anisotropy (FA) and time post-injury (post-acute and chronic) entered as main effects. One-way tests were computed if significant DTI metric × time post-injury interactions were present. All participants were included in these analyses.

## Results

**FIG. 1.**
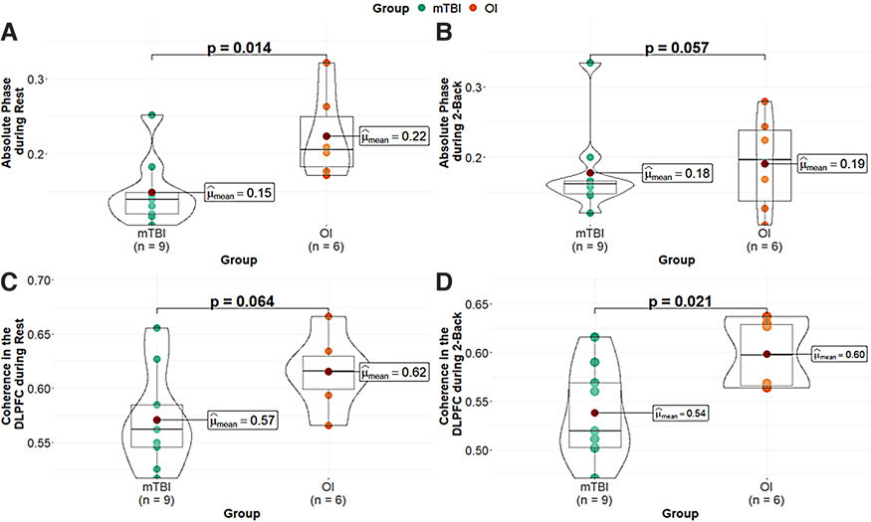
Group differences between OI and mTBI using fNIRS. Data are shown for absolute phase during rest (**A**) and 2-back (**B**) and for DLPFC coherence at rest (**C**) and during the 2-back (**D**). DLPFC, dorsolateral pre-frontal cortex; fNIRS, functional near-infrared spectroscopy; mTBI, mild traumatic brain injury; OI, orthopedic injury.

Using the mean over all connections, we observed significant differences between injury groups, in absolute phase of the hemodynamic oscillations during the rest condition (*F*_(1, 13)_ = 8.07, *p* = 0.014; Fig. 1A). The mTBI group showed decreased absolute phase in comparison to the OI group. The group difference in absolute phase during the 2-back (*F*_(1, 13)_ = 4.37, *p* = 0.057, η^2^ = 0.25; Fig. 1B) was not significant, but the direction and magnitude of effect was similar, suggesting that absolute phase was lower in mTBI relative to OI.

Group differences in coherence during the rest condition were not significant (*F*_(1, 13)_ = 4.09, *p* = 0.064). However, significant group differences were also observed during the 2-back condition (Fig. 1D; *F*_(1, 13)_ = 6.92, *p* = 0.021, η^2^ = 0.35), with lower DLPFC coherence in the mTBI group.

**FIG. 2.**
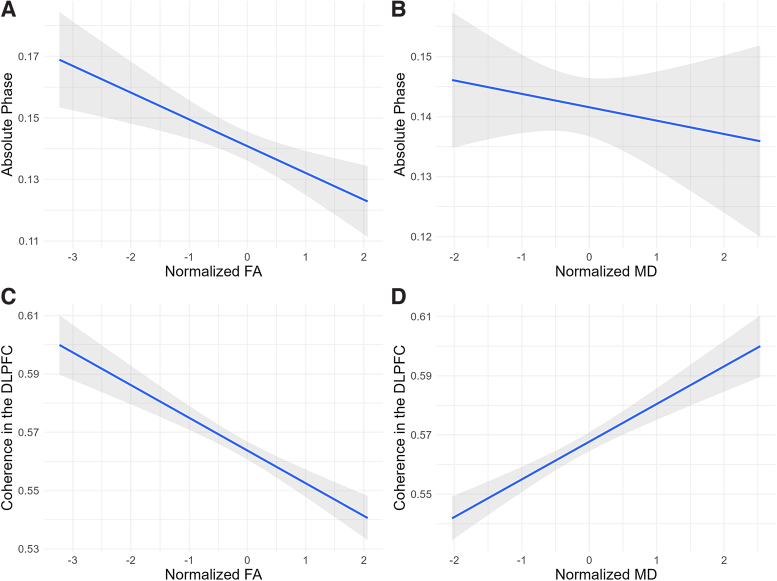
Association between global absolute phase during rest with (**A**) FA and (**B**) MD. Association of DLPFC coherence during the 2-back with (**C**) FA and (**D**) MD. Shaded gray areas denote the 95% confidence intervals. Data points were omitted for clarity. DLPFC, dorsolateral pre-frontal cortex; FA, fractional anisotropy; MD, mean diffusivity.

When examining the association between fNIRS and DTI metrics, we focused on the fNIRS parameters that showed significant group differences. Thus, both absolute phase during rest as well as coherence in the DLPFC during the 2-back were entered into the DTI analyses. Slopes for each model are shown in Figure 2. In all models, effects of time post-injury were not significant.

FA (*F*_(1, 122)_ = 5.29, *p* = 0.023, η^2^ = 0.04; Fig. 2A) was significantly and negatively related to absolute phase. Increased coherence in the DLPFC was associated with lower FA (*F*_(1, 122)_ = 10.10, *p* = 0.002, η^2^ = 0.08; Fig. 2C) and higher MD (*F*_(1, 122)_ = 12.14, *p* < 0.001, η^2^ = 0.09; Fig. 2D).

## Discussion

### Part 1: Group differences in functional near-infrared spectroscopy metrics

Group differences in hemodynamic function have shown promise as a biomarker after brain injury.^26,27^ Here, we showed that functional connectivity of the hemodynamic signal between brain regions measured using these fNIRS metrics distinguished pediatric mTBI from mild OI. Absolute phase distinguished groups across all connections. Decreased coherence in the bilateral DLPFC was present during the 2-back and showed a similar difference, albeit non-significant, during the rest condition. Finally, altered fNIRS metrics related to DTI metrics, across groups.

The reduced coherence observed is consistent with previous findings post-mTBI in adults.^8^ This reduction may relate to changes in neuronal activity, vascular regulation, or reduced interhemispheric communication attributable to injury in associated fiber tracts.^28,29^

We also observed group differences in the absolute phase of coherence. To our knowledge, this is the first report of hemodynamic phase as a potential marker of brain injury. Findings with this metric were global and not limited to a particular brain region. These results suggest that additional metrics beyond coherence can be used to assess functional brain connectivity.

The region selected for functional connectivity assessment may impact the ability to detect injury. Most studies to date on mTBI in children have focused solely on pre-frontal activity,^5–7,24,25^ but changes have also been noted in the motor cortex,^27^ whereas one study evaluated the frontal lobe in conjunction with the occipital lobe.^30^ Although differences are often observed in the DLPFC after mTBI, it remains to be determined whether measuring other regions will add to the differentiation of mTBI.

### Part 2: Relationship between functional near-infrared spectroscopy and diffusion tensor imaging metrics

Reduced coherence in mTBI related to differences in the white matter microstructure of interhemispheric pathways. White matter damage is related to the mechanical forces incurred during the concussive event.^28,29^ Here, fNIRS metrics (absolute phase and coherence) that distinguished groups also were associated with indices of white matter microstructure (i.e., FA and MD). Increasing values of both coherence in the DLPFC and absolute phase globally were associated with decreasing FA (Fig. 2). This suggests that fNIRS metrics assessing functional communication between brain regions relate to changes in underlying white matter microstructure that may occur with mTBI.

### Limitations

The current sample size limited statistical power and also prevented further examination of whether the relationship between fNIRS and DTI metrics differed by group or sex. We did use the full statistical power of 226 participants (the Calgary cohort of the A-CAP study) to normalize DTI metrics. In analyses of the whole multi-center DTI data set (*n* = 560), only one sex-related difference in any DTI metric (MD) was found and the effect was small.^4^ A larger sample would enable more in-depth investigation into moderators (e.g., group, region-of-interest, and age- and sex-related effects) present post-mTBI.^22^

The clinical significance of the differences in white matter microstructure is poorly understood in youth with mTBI and beyond the scope of this study. This study did not evaluate the diagnostic or prognostic significance of the observed findings (e.g., changes in white matter microstructure and/or decreases in absolute phase with fNIRS).

## Conclusion

These results suggest that fNIRS shows promise as a biomarker of concussion. Interhemispheric coherence in the DLPFC successfully differentiated groups, consistent with previous studies. However, this is the first study to demonstrate that global functional connectivity (i.e., absolute phase) also can distinguish mTBI from OI in children.

We also showed a negative association of fNIRS coherence and absolute phase with FA on DTI. These relationships support the hypothesis that microstructural integrity may relate to brain function. The findings suggest the potential complementarity of fNIRS and DTI and their shared potential for assessing mTBI in children.

## Abbreviations Used

DLPFC: dorsolateral pre-frontal cortex
DTI: diffusion tensor imaging
FA: fractional anisotropy
fNIRS: functional near-infrared spectroscopy
M: mean
MD: mean diffusivity
MRI: magnetic resonance imaging
mTBI: mild traumatic brain injury
OI: orthopedic injury
SD: standard deviation
TBI: traumatic brain injury
